# Utility of Computed Tomographic Angiography for Pulmonary Hypertension Assessment in a Cohort of West Highland White Terriers With or Without Canine Idiopathic Pulmonary Fibrosis

**DOI:** 10.3389/fvets.2021.732133

**Published:** 2021-09-23

**Authors:** Eugénie Soliveres, Kathleen Mc Entee, Thierry Couvreur, Aline Fastrès, Elodie Roels, Anne-Christine Merveille, Alexandru-Cosmin Tutunaru, Cécile Clercx, Géraldine Bolen

**Affiliations:** ^1^Department of Clinical Sciences, Companion Animals, Faculty of Veterinary Medicine, Fundamental and Applied Research for Animals & Health (FARAH), University of Liège, Liège, Belgium; ^2^Laboratory of Physiology and Pharmacology, Faculty of Medicine, Université Libre de Bruxelles, Brussels, Belgium; ^3^Department of Radiology, Christian Hospital Center Liège, Liège, Belgium

**Keywords:** computed tomography angiography, pulmonary hypertension, pulmonary trunk, canine idiopathic pulmonary fibrosis, West Highland white terrier

## Abstract

West Highland white terriers (WHWTs) affected with canine idiopathic pulmonary fibrosis (CIPF) are at risk of developing precapillary pulmonary hypertension (PH). In humans, thoracic computed tomography angiography (CTA) is commonly used to diagnose and monitor patients with lower airway diseases. In such patients, CTA helps to identify comorbidities, such as PH, that could negatively impact prognosis. Diameter of the pulmonary trunk (PT), pulmonary trunk-to-aorta ratio (PT/Ao), and right ventricle-to-left ventricle ratio (RV/LV) are CTA parameters commonly used to assess the presence of PH. Pulmonary vein-to-right pulmonary artery ratio (PV/PA) is a new echocardiographic parameter that can be used in dogs to diagnose PH. The primary aim of this study was to evaluate the use of various CTA parameters to diagnose PH. An additional aim was to evaluate the correlation of RV/LV measurements between different CTA planes. CTA and echocardiography were prospectively performed on a total of 47 WHWTs; 22 affected with CIPF and 25 presumed healthy control dogs. Dogs were considered to have PH if pulmonary vein-to-right pulmonary artery ratio (PV/PA) measured on 2D-mode echocardiography was less than to 0.7. WHWTs affected with CIPF had higher PT/Ao compared with control patients. In WHWTs affected with CIPF, PT size was larger in dogs with PH (15.4 mm) compared with dogs without PH (13 mm, *p* = 0.003). A cutoff value of 13.8 mm predicted PH in WHWTs affected with CIPF with a sensitivity of 90% and a specificity of 87% (AUC = 0.93). High correlations were observed between the different CTA planes of RV/LV. Results suggest that diameter of the PT measured by CTA can be used to diagnose PH in WHWTs with CIPF.

## Introduction

Canine idiopathic pulmonary fibrosis (CIPF) of the West Highland white terrier (WHWT) is a progressive and fatal interstitial pulmonary disease (1, 2). CIPF shares clinical and pathologic features with human idiopathic pulmonary fibrosis (IPF) and other interstitial pulmonary diseases (1, 3). Affected WHWTs are middle aged to old with no sex predisposition (1, 2, 4). Most common clinical signs include exercise intolerance and cough (1, 2, 4). Dogs can also present dyspnea, tachypnea, cyanosis, and collapse (1, 3). The diagnosis is based on signalment, duration and evolution of symptoms, clinical examination, results of diagnostic imaging especially high-resolution computed tomography (HRCT) and, because pulmonary biopsies are not routinely performed, exclusion of other respiratory diseases (1, 4).

One of the main complications of CIPF is the development of precapillary pulmonary hypertension (PH) with a percentage of affected dogs varying between 40 and 60% (2, 3, 5, 6). PH is defined as an abnormally increased pulmonary arterial pressure, >46 mmHg for systolic pressure (7, 8). PH is of precapillary origin and classified to proposed group 3 in the American College of Veterinary Internal Medicine (ACVIM) consensus statement, including pulmonary hypertension secondary to respiratory disease, hypoxia, or both (8). The gold standard for PH diagnosis in human IPF is right heart catheterization, and PH has been defined as a mean pulmonary arterial pressure ≥25 mmHg at rest (9). In veterinary medicine, right heart catheterization is not carried out routinely and the probability that a dog has PH is largely based on echocardiographic signs of PH and on estimation of pulmonary arterial pressure via Doppler echocardiographic measurement of tricuspid regurgitation pressure gradient (TRPG) (8, 10). Calculation of pressure gradients by measuring tricuspid or pulmonary regurgitation velocity is not always feasible and accurate because of the poor acoustic window, especially in patients with chronic lung disease. Moreover, laborious breathing may impair proper Doppler beam alignment (11–14). In the absence of reliably measurable tricuspid or pulmonary regurgitation, indirect indices of PH have been developed (15). The ACVIM consensus statement to diagnose PH in dogs defined the echocardiographic criteria used to determine the probability of PH by a combination of TRPG and appearance of anatomic echocardiographic sites such as ventricles, pulmonary artery, right atrium, and caudal vena cava (8). Recently, a value <0.7 for the pulmonary vein-to-right pulmonary artery ratio (PV/PA) measured on 2D-mode echocardiography was demonstrated to be one of the most accurate surrogates for diagnosis of moderate to severe precapillary PH (defined as a tricuspid regurgitation peak gradient superior to 50 mmHg) (16).

In human medicine, HRCT is commonly used in patients with lower airway disease (17), and in addition to specifying the cause of the disease, HRCT with computed tomographic angiography (CTA) can identify comorbidities with repercussions on prognosis such as PH (18). In humans, diameter of the pulmonary trunk (PT), pulmonary trunk-to-aorta ratio (PT/Ao), and right ventricle-to-left ventricle ratio (RV/LV) are measured alone or in combination to assess the presence of PH (19–21). In veterinary medicine, HRCT provides superior evaluation of the lung compared with conventional radiographs and has been proven useful in diagnosing CIPF (3, 4, 22). The assessment of CTA indices of PH during HRCT might improve early detection of PH in WHWTs affected with CIPF. Therefore, if thoracic HRCT allowed a proper estimation of PH, there would be no need for repeated echocardiography in the follow-up of dogs with chronic interstitial (group 3 PH) diseases.

The primary aim of the study was to evaluate the diagnostic accuracy of PT, PT/Ao, RV/LV, and PV/PA measured by CTA to predict PH in a population of middle-aged to old WHWTs affected or not with CIPF with PH defined as PV/PA <0.7 measured on 2D-mode echocardiography. We hypothesized that, like in human medicine, PT and PT/Ao measured by CTA are more useful to differentiate dogs with or without PH than PV/PA and RV/LV. A secondary aim was to evaluate the correlation of RV/LV measurements between different CTA planes.

## Materials and Methods

### Dogs

The study design was prospective, observational, and case-control. WHWTs with clinical signs of CIPF and clinically healthy middle-aged to old WHWTs were prospectively enrolled at the Veterinary Teaching Hospital of the University of Liège from March 2013 to October 2019 by way of the canine idiopathic pulmonary fibrosis project (http://www.caninepulmonaryfibrosis.ulg.ac.be). All procedures were approved by the Committee of Experimental Animals of the University of Liège, Belgium (permit number: 1435, date of approval: April, 2013; permit number: 1649, date of approval: January 2015; and permit number: 2245, date of approval: April 2020) and performed with the signed consent of the owners.

WHWTs were considered to have CIPF if they had medical history and clinical signs consistent with the disease (cough, exercise intolerance, respiratory distress, and/or crackles on lung auscultation), and compatible thoracic imaging findings on HRCT (22). Exclusion criterion was a concomitant left- or right-sided cardiac disease. WHWTs were considered a control if they did not have history of cardiovascular or respiratory signs, if echocardiography did not show any primary cardiac disease, and if clinical examination and pulmonary images on HRCT were within normal limits. A 6-min walking test and endoscopy with bronchoalveolar lavage were performed in the majority of dogs in the CIPF group. In all dogs, thoracic HRCT scan with CTA and 2D-mode echocardiography were performed the same week. The dogs were subsequently divided into four groups (control without PH, control with PH, CIPF without PH, CIPF with PH) depending on the presence or not of PH at 2D-mode echocardiography. Dogs were considered to have PH if PV/PA measured on 2D-mode echocardiography was <0.7 (16).

### Echocardiographic Measurements

Transthoracic 2D was performed, using two different ultrasound machines, one^1^ with 2.2–3.5 and 5.5–7.5 MHz phased-array transducers between 2013 and 2018, and the other^2^ with 2.4–8 and 4–12 MHz phased-array transducers since 2018. Dogs were placed in right and left lateral recumbency, and a simultaneous ECG was recorded. As previously described, inner diameters of the pulmonary vein and right pulmonary artery were measured in 2D at the end of the T wave in a right parasternal long-axis view optimized to visualize the heart base, and their ratio PV/PA was calculated (16).

### Thoracic Computed Tomography Angiography Acquisition

Thoracic CTA was performed in sternal recumbency, under general anesthesia after a transient period of hyperventilation to induce apnea and avoid motion artifacts. Dogs were sedated with butorphanol (0.3–0.4 mg/kg), and anesthesia was induced with propofol. Dogs were maintained on general anesthesia with isoflurane gas and 100% oxygen.

Two different CT scanners were used: a 16-multislice CT scanner^3^ between 2013 and April 2019 and a 64-multislice CT scanner^4^ between April 2019 and October 2019. Acquisition parameters used were as follows for the 16-multislice CT scanner: tube voltage 120 kV, reference tube current 130 mA, and pitch 0.7–1.15. Scan tube current was modulated by automatic exposure control (Care Dose, Siemens Medical Solutions, International). Image data sets were reconstructed using parameters of 200–300 mm field of view, 512 × 512 matrix, 1 mm slice thickness, and Br-30f medium smooth reconstruction algorithm (window level 40 and window width 400) with filter back projection. Acquisition parameters for the 64-multislice CT scanner were as follows: tube voltage 120 kV, reference tube current 170 mA, and pitch 1.2. Scan tube current was modulated by automatic exposure control (Care Dose, Siemens Medical Solutions, International). Image data sets were reconstructed using parameters of 300 mm field of view, 512 × 512 matrix, 1 mm slice thickness and Br-36 mediastinum reconstruction algorithm (window level 55 and window width 360) with iterative reconstruction.

Post-contrast images were acquired directly after manual injection of 2 ml/kg of iodinated contrast medium (iohexol^5^ or iopromide^6^).

### Thoracic Computed Tomography Angiography Measurements

**Figure 1:**
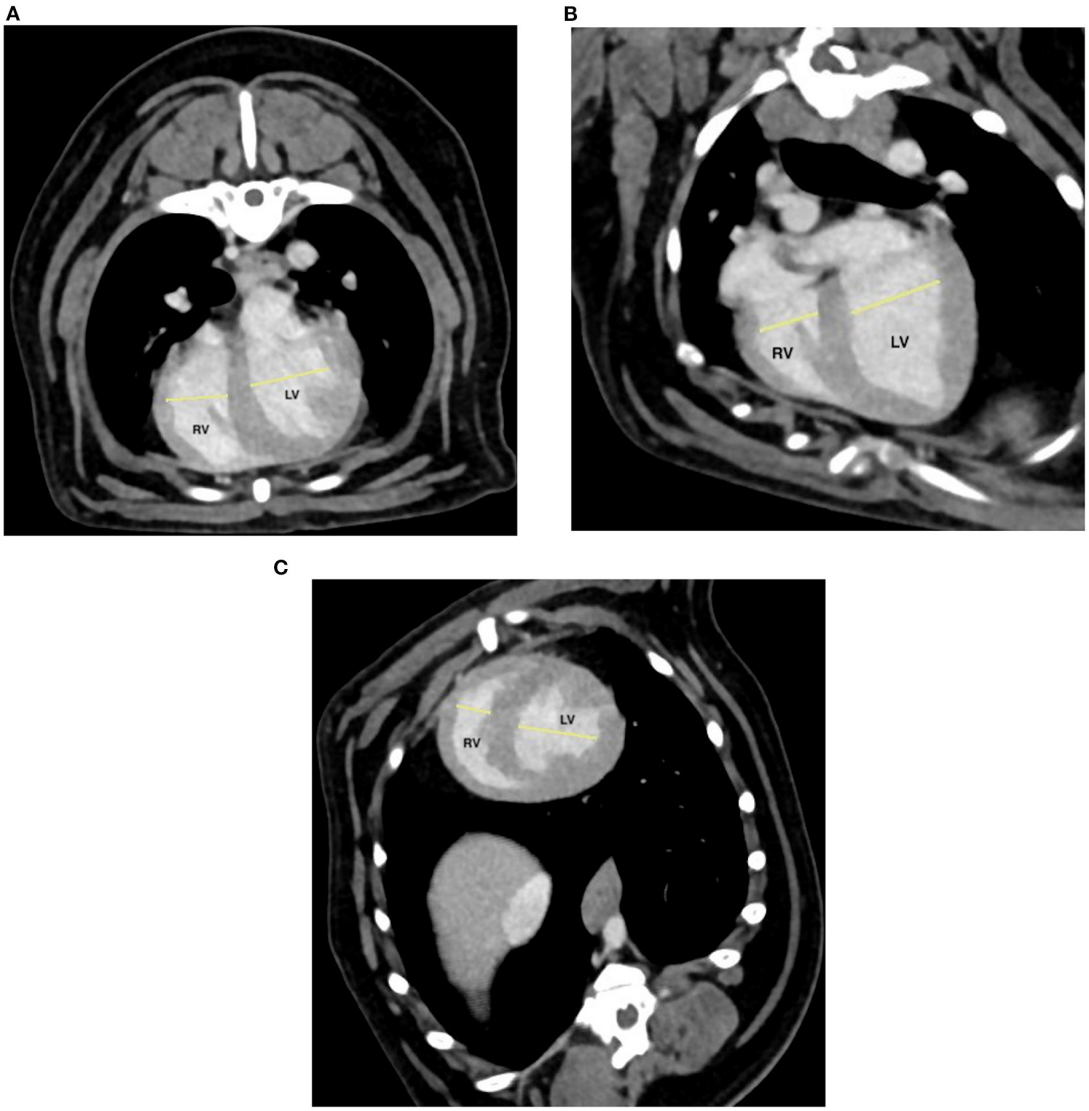
Post-contrast transverse **(A)** and manually reformatted **(B,C)** thoracic computed tomographic angiography (CTA) at the level of the ventricles. **(B)** Standard four-chamber echocardiographic view (4C); **(C)** standard short-axis echocardiographic view (SA). Right ventricle (RV) and left ventricle (LV) were measured at their largest diameter.

**Figure 2:**
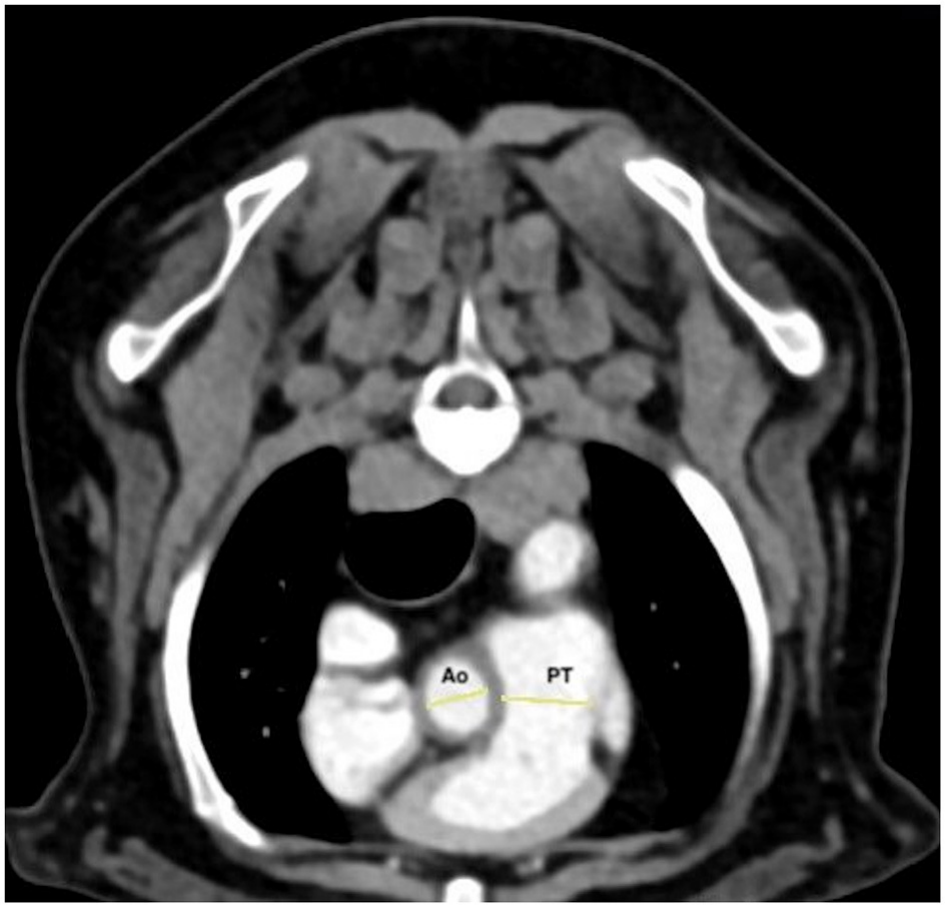
Post-contrast transverse thoracic computed tomographic angiography (CTA) at the level of the pulmonary trunk. The pulmonary trunk (PT) was assessed just ventral to the division of the pulmonary arteries, and the ascending aorta (Ao) was assessed on the same image.

**Figure 3:**
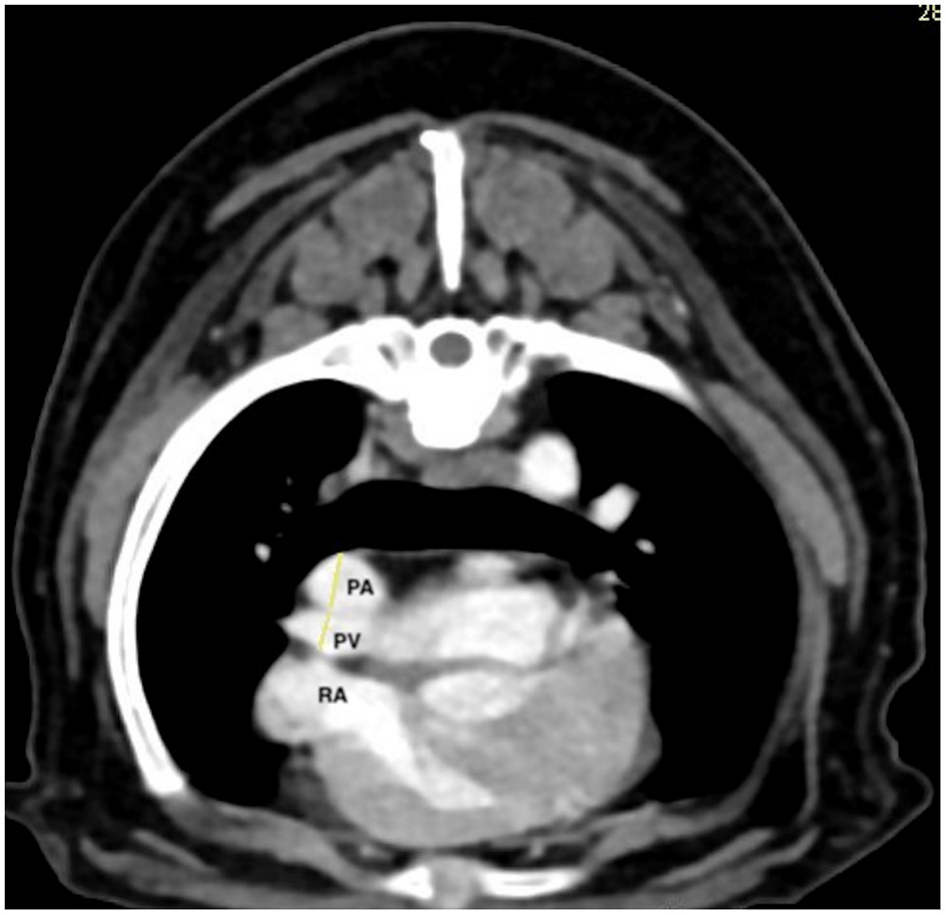
Post-contrast transverse thoracic computed tomographic angiography (CTA) at the level of the right atrium (RA). The right pulmonary artery (PA) and its adjacent pulmonary vein (PV) were measured dorsal to the right atrium. The pulmonary artery is dorsal and the vein ventral.

All images were blindly reviewed twice by one radiologist (TC) with a minimum of 3 weeks between each reviewing, to obtain a mean value for each measure. All measurements were performed using the methods described by Couvreur et al. (23). Right- and left-ventricle diameters were obtained from acquired transverse (T) images and on transverse reformatted images which was equivalent to the standard four-chamber echocardiographic view (4C) and on a dorsal-reformatted images equivalent to the standard short-axis echocardiographic view (SA) (Figures 1A–C). The right ventricle and left ventricle were measured at their largest diameter on the same axis to calculate the RV/LV. Diameters of PT and aorta and of the right pulmonary artery and its adjacent pulmonary vein were measured on acquired transverse images. The PT was assessed just ventral to the division of the pulmonary arteries. The ascending aorta was assessed on the same image (Figure 2). The right pulmonary artery and its adjacent pulmonary vein were measured dorsal to the right atrium (Figure 3). The PT/Ao and PV/PA on CTA were calculated.

### Statistical Analysis

Statistical analyses were performed using a commercially available software^7^ (Excel, Microsoft Office; and XLStat software; Addinsoft SARL, International). Statistical significance was set at *p* < 0.05. Proportions were compared by a chi-squared test. A Shapiro-Wilk test was applied to assess to the normality of distribution of continuous variables. A Pearson correlation test was used to assess the strength of the relationship between RV/LV measured in the different CTA planes. Correlation was defined as high when *r* was between 0.7 and 0.9, moderate when *r* was between 0.5 and 0.7, and weak when *r* was inferior to 0.5. Comparisons between groups were performed using Student's *t*-test or one-way analysis of variance followed by pairwise comparisons using a Dunn's test with Bonferroni correction (corrected *p* < 0.008). Receiver operator characteristic (ROC) curve analysis was performed to determine the optimal cutoff value of PT for the prediction of PH in CIPF dogs defined by PV/PA measured on 2D echocardiography <0.7.

## Results

### Dogs

Forty-seven WHWTs were included in the study, 22 with CIPF (12 CIPF without PH and 10 CIPF with PH) and 25 control dogs (22 control dogs without PH and three control dogs with PH). Dogs with CIPF were older than control dogs (11.2 years ± 2 vs. 9.9 years ± 2.3, *p* = 0.041). No statistical difference in sex was observed between the groups (*p* = 0.210), 11 of the 19 females were classified as having CIPF, and 11 of the 28 males were classified as having CIPF. Concomitant comorbidities in the control group included atopy in three dogs, urinary incontinence in two dogs, chronic otitis externa and facial neuropathy in one dog, chronic otitis externa in one dog, coxo-femoral luxation in one dog, rectal mass in one dog, nasal tumor in one dog, and allergic dermatitis in one dog. Concomitant comorbidities in CIPF group included atopy in two dogs; otitis in one dog; otitis and atopy in one dog; unclassified dermatitis in one dog; cataract and unclassified cutaneous disease in one dog; Cushing disease, diabetes mellitus, and degenerative joint disease in one dog; Cushing disease and rupture of cranial cruciate ligaments in one dog; bilateral otitis, chronic kidney disease, uveitis, kerato-conjunctivitis sicca, and rhinitis in one dog; and interdigit abscess in one dog.

### Clinical Data

In CIPF WHWTs, the mean duration of clinical signs was 9.42 ± 12.07 months. The most reported clinical signs were cough (*n* = 18) and exercise intolerance (*n* = 18). Dyspnea was present in 10 dogs during clinical examination and cyanosis in two dogs. Crackles were heard in auscultation in 21 CIPF dogs.

Bronchoscopy was performed in 31 dogs, including 21 CIPF and 10 control dogs, and revealed grade 1 tracheal collapse in seven CIPF dogs and five control dogs, grade 2 tracheal collapse in six CIPF dogs and grade 3 tracheal collapse in two CIPF dogs (*p* = 0.244), bronchomalacia in 12 CIPF dogs and two control dogs (*p* = 0.052), and mucus in the bronchi in 10 CIPF and one control (*p* = 0.041). No control dog with PH had either tracheal collapse or bronchomalacia.

PH, defined by a PV/PA <0.7 measured in 2D-mode echocardiography, was more frequently observed (*p* = 0.004) in the CIPF group (10/22, 45.5%) than in the control group (3/25, 12%). Tricuspid regurgitation was observed in 12/22 CIPF and eight of 25 control dogs (*p* = 0.344). The mean tricuspid regurgitation peak gradient was 39.5 ± 12.9 in CIPF dogs and 30.7 ± 11.5 in control dogs (*p* = 0.173).

At the time of diagnosis, six control dogs and 10 CIPF dogs were under therapy. In the control group, treatment included prednisolone (0.7 mg/kg SID) in one dog, piroxicam (0.3 mg/kg SID) in one dog, pimobendan (0.13 mg/kg BID) in one dog, inhalation of acetylcystein BID in one dog, and cefovecin in one dog. One dog received interferon. Treatments in the CIPF group were theophylline (8.8–11 mg/kg BID in two dogs and unknown dosage in one dog), sildenafil (0.7–1 mg/kg BID) in two dogs, pimobendan (0.20 mg/kg BID) in one dog, codeine (1 mg/kg in one dog and unknown dose in one dog), prednisolone (1 mg/kg BID in one dog, 0.6 mg/kg SID in one dog, 0.25 mg/kg every 2 days in one dog, unknown dose in one dog), budesonide (0.15 mg/kg BID) in one dog, benazepril (0.7 mg/kg SID) in one dog, and propentofylline (5.5 mg/kg BID) in one dog. One dog received furosemide and amoxycillin/clavulanic acid at unknown dosage and another dog received ursodeoxycholic acid, trilostane, and insulin.

### Thoracic HRCT

Regarding pulmonary lesions observed in CIPF dogs, ground glass opacity was observed in 22 of the 22 dogs. Other findings included mosaic attenuation pattern in 20 dogs, areas of consolidation in 10 dogs, parenchymal bands in nine dogs, ground glass nodules in six dogs, and bullae in two dogs. Tracheal collapse was classified into mild, moderate, or severe (3). Tracheal collapse was observed in 18 dogs, eight with mild to moderate collapse and 10 with severe tracheal collapse. Bronchial collapse was visualized in nine dogs, bronchial dilation in three dogs, thickening of the bronchial wall in three dogs, and saccular bronchi in four dogs.

In control dogs, eight of the 20 dogs had mild to moderate tracheal collapse including one dog in the control group with PH.

### CTA

#### Comparison of CTA-Derived PH Indices Between Control and CIPF Dogs With or Without PH

**Figure 4:**
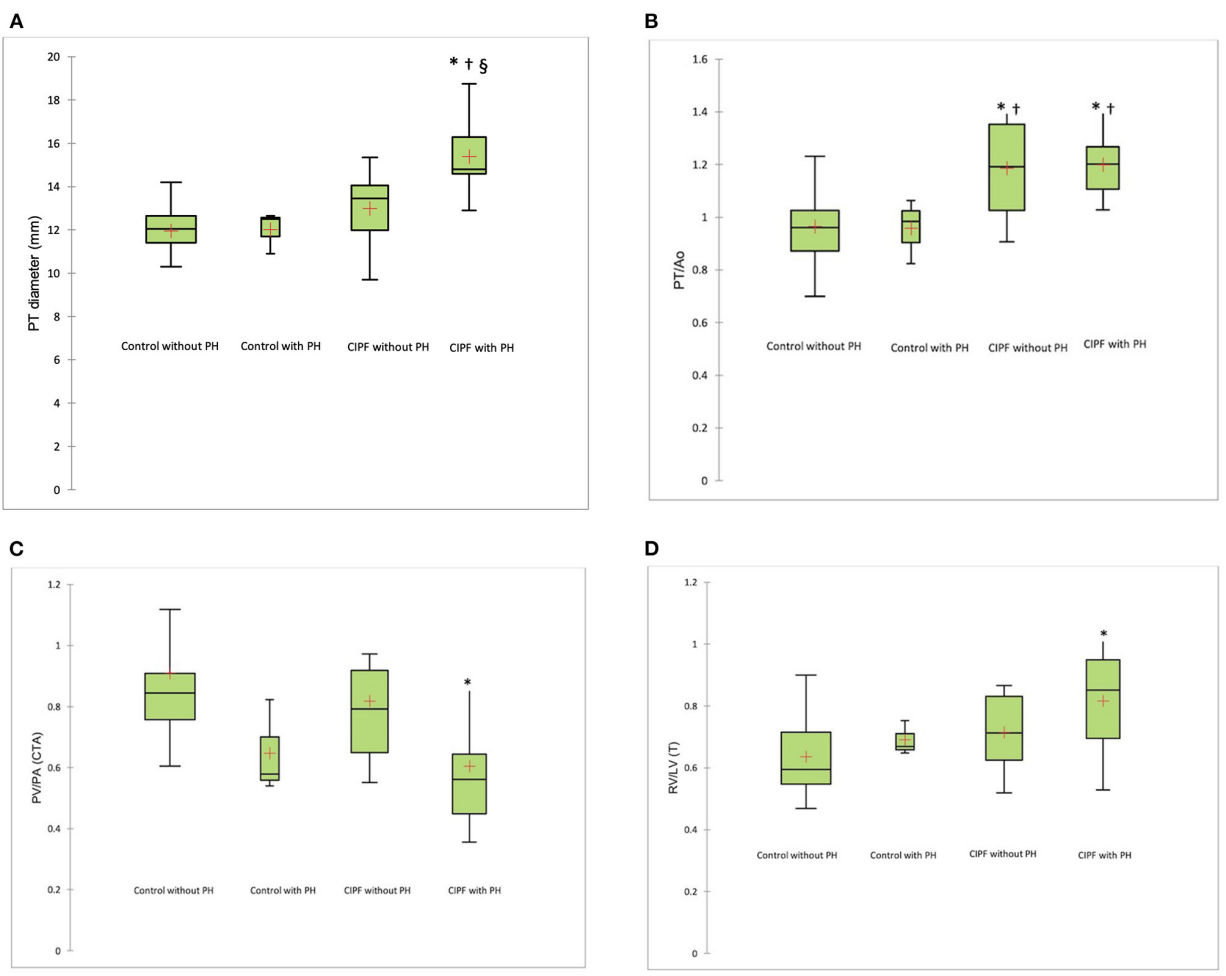
Box plot illustrating PT **(A)**, PT/Ao **(B)**, PV/PA measured on CTA **(C)**, and RV/LV (T) **(D)** according to groups. The whiskers indicate the range of values. The box contains the 25th−75th centiles. The line within the box indicates the median, and the cross indicates the mean. *p < 0.008, significantly different from control without PH; ^†^*p* < 0.008, significantly different from control with PH; ^§^*p* < 0.008, significantly different from CIPF group without PH. PT, pulmonary trunk; PT/Ao, pulmonary trunk-to-aorta ratio; PV/PA, pulmonary vein-to-right pulmonary artery ratio; CTA, computed tomographic angiography; RV/LV (T), right ventricle-to-left ventricle ratio measured on CTA acquired transverse plane; PH, pulmonary hypertension; CIPF, canine idiopathic pulmonary fibrosis.

WHWTs affected by CIPF with a comorbidity of PH had higher mean PT diameter compared with the three other groups (Figure 4A, *p* = 0.003). Area-under-the receiver-operating characteristic curve was 0.93. A cutoff value of >13.8 mm predicted PH in CIPF dogs with a sensitivity of 90% and a specificity of 87%.

The CIPF dogs with or without PH had higher PT/Ao ratio compared with control dogs with or without PH (Figure 4B, *p* < 0.008). PV/PA was decreased in the CIPF with PH group compared with control group without PH (Figure 4C, *p* = 0.004). RV/LV(T) was higher in CIPF dogs with PH than in control dogs without PH (Figure 4D, *p* = 0.001). A similar observation was noted for RV/LV (SA) (0.816 ± 0.109 vs. 0.665 ± 0.065, *p* < 0.0001) and RV/LV (4C) (0.847 ± 0.137 vs. 0.697 + 0.08, *p* = 0.006), with RV/LV higher in CIPF dogs with PH than in control dogs without PH.

#### Correlations Between the Different CTA Planes Used to Measure the RV/LV

The RV/LV in CTA was highly correlated between the three CTA planes [*r* = 0.755, *p* < 0.001, between RV/LV (T) and RV/LV (4C), *r* = 0.797, *p* < 0.001, between RV/LV (T) and RV/LV (SA) and *r* = 0.881, *p* < 0.001, between RV/LV (4C) and RV/LV (SA)].

## Discussion

Results of the present study confirmed partially our hypothesis. In a population of WHWTs with PH, PT diameter measured by CTA was higher in CIPF WHWTs affected with PH compared with CIPF WHWTs without PH and control WHWTs. Using a cutoff value of 13.8 mm, PT diameter differentiated CIPF dogs with PH from other groups with a high diagnostic accuracy. The PT/Ao ratio was higher in CIPF dogs whether or not there was PH.

In human medicine, the gold standard to diagnose an increase in pulmonary arterial pressure is right heart catheterization (24). In veterinary medicine, this technique is not commonly used and echocardiography remains the method of choice to diagnose PH (15). During systole, right ventricular and pulmonary pressures are similar. Applying the Bernoulli equation, the systolic pulmonary pressure is classically extrapolated from the velocity of the tricuspid regurgitation measured by ultrasonography. However, tricuspid regurgitation is not always easily and accurately measurable because of a poor alignment of the Doppler cursor with the direction of the tricuspid regurgitation flow or because of a poor acoustic window, especially when concurrent pulmonary disease is present. Some studies in human medicine have compared the accuracy of transthoracic echocardiography with assess TRPG in patient suffering from PH with or without severe pulmonary disease, including IPF (11, 25, 26). Estimation of the systolic pulmonary artery pressure was inaccurate in 40–52% of cases depending on studies. Arcasoy et al. observed that estimation of the systolic pulmonary artery pressure was not possible in 56% of patient with advanced lung disease. Consequently, other echocardiographic parameters can be used to assess the presence or not of PH. The ACVIM consensus statement recently defined guidelines to determine the probability of PH in dogs (8). The presence of a tricuspid regurgitation >3.4 m/s corresponds to an intermediate probability of PH. When no tricuspid regurgitation is observed or when it is not measurable, assessment of the appearance of anatomic echocardiographic sites, such as ventricles, pulmonary artery, right atrium, and caudal vena cava, was recommended (8). In the present study however, we selected dogs with PH based on a value of PV/PA <0.7 at 2D-mode echocardiography. This decision, taken before the ACVIM consensus statement was published, can be justified as follows. PV/PA is an echocardiographic parameter that has recently been described in veterinary medicine to evaluate the presence of PH (6, 16, 27). The normal value of this PV/PA has been defined to be ~1 (27). The usefulness of echocardiographic measurement of PV/PA to diagnose PH has been assessed in a cohort of dogs suffering from precapillary PH. Roels et al. found that PV/PA measured on 2D-mode and motion-mode proportionally decreased when PH increased, due to increased PA diameter and decreased PV diameter. A cutoff value of 0.7 for PV/PA measured on 2D-mode echocardiography was proven to diagnose dogs with moderate to severe precapillary PH with a good sensitivity (96%) and specificity (82%) (16). Moreover, a recent study highlighted the usefulness of PV/PA measured with 2D-mode echocardiography as a non-invasive method to diagnose PH in a population of WHWTs with or without PH (6).

PH is a common comorbidity of CIPF in WHWTs. In this study, 45% of WHWTs affected with CIPF had PH according to PV/PA measured on 2D-mode echocardiography. This proportion is in accordance with previously reported prevalence of 44% according to tricuspid regurgitation velocity in a cohort of 45 WHWT affected with CIPF (5) and of 60% according to PV/PA measured on 2D-mode echocardiography in a cohort of 25 CIPF WHWT (6). In human patients with interstitial lung disease, the presence of PH is associated with a worse prognosis (28, 29). A same observation has been made in a population of dogs with various respiratory disease and concomitant PH (30). Accordingly, early diagnosis can optimize treatment and potentially improves survival and quality of life (28, 29). Several studies support the use of thoracic HRCT as the diagnostic imaging of choice for characterizing CIPF in WHWT (3, 4). With the development of teleradiology services, veterinary clinics equip themselves with a CT scanner and routinely performed thoracic CT to explore pulmonary diseases, even if no diplomate radiologist is available on site. Performing a standard non-ECG-gated CTA confers the opportunity to identify PH during one HRCT-CTA procedure even in dogs with no clinical suspicion of PH.

In human medicine, an increased diameter of the PT and higher PT/Ao are intensively studied features to predict PH on CTA (19, 31–34). A PT diameter higher than 29 mm is in favor of PH (31). To the best of our knowledge, it is the first time that PT diameter alone is tested as a CTA index of PH in dogs. In our study, PT diameter was larger in the CIPF dogs with PH compared with the three other groups. In this cohort, a cutoff value of >13.8 mm predicted PH in CIPF dogs with a sensitivity of 90% and a specificity of 87%. This result suggests that PT diameter measured on CTA may be a useful additional index to aid in the detection of PH in WHWT affected by CIPF. As the study involved a unique breed, the WHWT, the absolute value of PT could be used without normalization for dog size.

In human patients with chronic obstructive pulmonary disease, a PT/Ao higher than 1 on CTA was associated with a worse prognosis compared with patients with a lower PT/Ao (35). In our study, the mean PT/Ao of 0.97 ± 0.12 obtained for our 22 control WHWT without PH was close to the 1.03 ± 0.08 ratio published in 10 dogs without PH using the expiratory contrast protocol (36). On the other hand, our results differ from the 1.26 ± 0.11 value reported by Sutherland-Smith et al. in a population of dogs of various breeds, with or without PH (37). One explanation for this difference can be the landmark used to measure the aorta. Indeed, we measured the ascending aorta at the same level than the PT, while Sutherland-Smith et al. measured the descending aorta to calculate the PT/Ao. Another explanation can be the fact that only WHWT were included in our study while large-breed dogs were included in the previous study. In our study, PT/Ao was increased in the CIPF cohort whether or not PH was present. PT/Ao can be reliably used to detect PH in human patients with cardiopulmonary diseases (9, 38). However, results in patients with interstitial lung diseases are more conflicting. While some authors report that PT and PT/Ao are reliable predictors of PH in these patients (31), others have found that pulmonary artery dilatation occurs in the absence of PH in patients with lung fibrosis, which implies that such dilatation cannot be reliably used as a marker for PH (33, 34). They suppose that other mechanisms are implicated in the dilatation of the PT such as traction effect on the mediastinal vascular structures due to the restrictive lung physiology of IPF (32, 34). In a prospective study comparing the reliability of PT diameter and PT/Ao to predict PH in patients with or without interstitial lung disease, PT diameter was increased in PH patients in both groups, which suggests that PH does in fact increase PT diameter, regardless of underlying disease cause. Moreover, the ability of PT diameter to predict PH was superior to that of PT/Ao, but the accuracies of PT diameter and PT/Ao in the prediction of PH were overall lower in patients with interstitial lung disease than in those without interstitial lung disease (32). CIPF shares histopathological and imaging characteristics with both human usual interstitial pattern (e.g., human IPF) and non-specific interstitial pneumonia pattern (39). We can hypothesize that the traction effect could occur in CIPF and explain the increased PT/Ao in the CIPF without PH group. Confounding factors affecting the aortic diameter or amplification of the inaccuracies associated with the ratio of the two separate measures are other hypotheses to explain the discrepancy between the evolution of PT and PT/Ao.

Similarly, to a previous human study (40), we observed a high positive correlation between RV/LV in T, SA, and 4C views meaning that reformatted images are not necessary to assess this ratio. Whatever the view, RV/LV was higher in the CIPF group with PH compared with the three other groups. However, RV/LV stayed <1 and did not help in predicting PH in CIPF dogs. In humans, RV/LV on CTA may be used alone or in combination with PT/Ao to predict PH of different origins (20, 21). However, this ratio seems mostly used for risk stratification. Indeed, in a meta-analysis, RV/LV was a strong predictor of high mortality risk in cases of pulmonary thromboembolism (41), and in a recent study, a RV/LV >1 was an independent predictor of mortality or lung transplantation in patients with suspected interstitial lung disease and PH (42).

Conversely to PV/PA measured with echocardiography, CTA-derived PV/PA failed to predict PH in CIPF dogs. At each cardiac beat, there are physiologic cyclic changes of pulmonary vein and pulmonary artery diameters and PV/PA is measured at the end of the T wave during echocardiography (43). PH is also associated with a decrease in the right pulmonary artery distensibility (44). Therefore, an ECG-gated CTA could have decreased the variability of this measure. The position of the dog is also different between the two imaging methods. Indeed, during echocardiography, the dog is in right lateral recumbency while during CTA, the dog is in sternal recumbency. This difference could have an impact on PV compression and filling, pulmonary artery being dorsal and the vein ventral.

The main limitation of the present study was that pulmonary arterial pressure was not directly measured by right heart catheterization to assess the exact prevalence and severity of PH before the HRCT-CTA procedure. It would have been ideal to compare CTA and echocardiography results with those obtained using cardiac catheterization in order to help in highlighting any limitations of echocardiography and CTA for PH estimation. However, this technique was not chosen because of its inherent risk of complications. Echocardiography can be considered the method of choice to screen for PH. PV/PA in 2D-mode echocardiography was preferred to TRPG. Indeed, due to the lung disease and laborious breathing, tricuspid regurgitation pressure gradient was unmeasurable in a substantial number of dogs.

The fact that dogs were anesthetized during CTA is a second limitation but was mandatory and reflects real clinical practice. Anesthesia alters hemodynamics with potential repercussions on measures of CTA indices of PH that we tested. To reduce the variability due to anesthesia, the protocol was standardized and all dogs received butorphanol as a premedication agent. Butorphanol is known to be have limited hemodynamic effects compared with other sedatives (45, 46). Another limitation relates to the use of a 100% FiO_2_ during anesthesia. Oxygen could be toxic to the lungs when FIO_2_ higher than 60% is administered over 24 h at normal pressure (47). Toxicity of oxygen includes increase in atelectasis, oxidative stress to the lungs, and exacerbation of cancer effects (48). A high intraoperative FiO_2_ was associated with major respiratory complication within 7 days and with 30-day mortality (48). A high FiO_2_ during anesthesia may change the pulmonary vascular resistance (49). Oxygen is known to be a pulmonary vasodilator and could increase pulmonary flow by decreasing pulmonary vascular resistance (49) and then decreased pulmonary artery pressure (49). A study performed in various species found that hypoxic pulmonary vasoconstriction is weak in dogs (50) compared with other species. The response of pulmonary vasculature to hyperoxia however is not known and therefore a 100% Fi0_2_ could have changed the pulmonary artery pressure during the execution of the CT scans. During invasive pulmonary arterial pressure measurements, interventional cardiology procedures, or laser microlaryngeal surgery, human patients and especially pediatric patients are usually ventilated with ambient air (FiO_2_, 21%) (51–53). A high oxygen concentration is commonly used in pediatric patient to reduce pulmonary vascular resistance in conjunction with inhaled nitric oxide, when investigating pulmonary hypertension (51). In our study, we chose to use a 100% FiO_2_. Indeed, in the veterinary literature, 100% FiO_2_ is still commonly used during thoracic CTA procedure (36, 54–57). This protocol was selected when the present study started in 2013 and was maintained throughout the study to allow obtention of comparable measures of the heart and pulmonary vasculature.

Finally, using a cutoff value of 0.7 for PV/PA measured on 2D-mode echocardiography, three control dogs were classified as having PH. Tracheal collapse was noted in one of the three dogs only on HRCT and not on bronchoscopy. No parenchymal lesions were visualized on HRCT, but pulmonary biopsies were not performed. We cannot exclude that these dogs presented a preclinic stage of CIPF or another non-diagnosed cause of PH. On the other hand, PH was not confirmed by right heart catheterization. With a specificity of 0.82 for the PV/PA cutoff value measured on 2D-mode echocardiography used in this study, we cannot exclude some false-positive results (16).

In conclusion, results of the present study highlight the potential utility of PT diameter measured by CTA to predict PH in WHWT affected with CIPF. Further studies are needed to identify if the increase of PT and PT/Ao are PH and/or CIPF dependent.

## Data Availability Statement

The raw data supporting the conclusions of this article will be made available by the authors, without undue reservation.

## Ethics Statement

The animal study was reviewed and approved by Committee of Experimental Animals of the University of Liège, Belgium. Written informed consent was obtained from the owners for the participation of their animals in this study.

## Author Contributions

ES, KM, CC, and GB designed the study. ES wrote the manuscript. AF, ER, and CC recruited the dogs and collected clinical data. KM and A-CM performed and interpreted echocardiographies. ES, TC, and GB performed and interpreted HRCT. A-CT performed anesthesia of the dogs. TC, AF, KM, and ES compiled and analyzed the data. Results were interpreted, and the manuscript was critically revised by ES, KM, AF, CC, and GB. All authors read and approved the final manuscript.

## Conflict of Interest

The authors declare that the research was conducted in the absence of any commercial or financial relationships that could be construed as a potential conflict of interest.

## Publisher's Note

All claims expressed in this article are solely those of the authors and do not necessarily represent those of their affiliated organizations, or those of the publisher, the editors and the reviewers. Any product that may be evaluated in this article, or claim that may be made by its manufacturer, is not guaranteed or endorsed by the publisher.

## Footnotes

^1^Vivid I, General Electric Medical System, Machelen, Belgium.

^2^Vivid e95, General Electric Medical System, Machelen, Belgium.

^3^Siemens, Somatom 16, Erlangen, Germany.

^4^Siemens, Somatom Confidence 64, Erlangen, Germany.

^5^Omnipaque 300, General Electric Healthcare, Diegem, Belgium.

^6^Ultravist 300, Bayer Pharma, Diegem, Belgium.

^7^XLstat software, Addinsoft SARL, Paris, France.

## Abbreviations

4C: manually reformatted four-chamber cardiac view on computed tomographic angiography
ACVIM: American College of Veterinary Internal Medicine
CIPF: canine idiopathic pulmonary fibrosis
CTA: computed tomographic angiography
HRCT: high-resolution computed tomography
IPF: idiopathic pulmonary fibrosis
RV/LV: right ventricle-to-left ventricle ratio on CTA
PH: pulmonary hypertension
PT: pulmonary trunk
PT/Ao: pulmonary trunk-to-aorta ratio on CTA
PV/PA: pulmonary vein-to-right pulmonary artery ratio
SA: manually reformatted short-axis cardiac view on computed tomographic angiography
T: acquired transverse image on computed tomographic angiography
TRPG: tricuspid regurgitation pressure gradient
WHWT: West Highland white terrier.
